# From Policy to Practice: Bridging Sectors by Integrating Community Health and Collaborative Approaches to Enhance Neurocognitive Disorder Care

**DOI:** 10.1002/alz70858_103708

**Published:** 2025-12-26

**Authors:** Marie Christine Le Bourdais, Sonia BÉLANGER, Paolo Vitali, Felix Pageau, Ziad Nasreddine, Thomas Tannou, Guy Lacombe

**Affiliations:** ^1^ Alzheimer Society of Montreal, Montreal, QC, Canada; ^2^ Ministre des Ainés ‐ Ministère de la Santé et des Services sociaux, Quebec, QC, Canada; ^3^ The McGill University Research Centre for Studies in Aging, Montreal, QC, Canada; ^4^ Douglas Mental Health University Institute, Montreal, QC, Canada; ^5^ Université Laval, Québec, QC, Canada; ^6^ MoCA Clinic and Institute, Greenfield Park, QC, Canada; ^7^ CRIUGM, CIUSS Centre‐Sud de l'Ile de Montréal, Montreal, QC, Canada; ^8^ Sherbrooke University, Sherbbrooke, QC, Canada; ^9^ CIUSSS de l'Estrie‐CHUS, Sherbrooke, QC, Canada

## Abstract

A strong multisectoral approach leads to positive impacts across the life and care pathways for neurocognitive disorders. Community health organizations play a crucial role in supporting individuals with neurocognitive disorders and care partners, complementing clinical and policy‐driven approaches by providing grassroots support and adapted care. Aligning community health offerings with national policies, research and clinical practices is essential to create a cohesive, adapted, integrated, responsive, and person‐centered care system.

The Alzheimer Society of Montreal spearheaded the creation of a think tank, recognizing the need for a unified effort to tackle the multifaceted issues surrounding neurocognitive disorders. Bringing together diverse experts ensures that multiple perspectives are considered, leading to more comprehensive and effective solutions. These efforts empower communities and partners, providing them with the tools and support needed to actively participate in and benefit from collaborative initiatives and learnings, as well as creating bridges between knowledge and interventions.

As part of the care ecosystem, community health organizations promote early detection through awareness campaigns and education, ensuring that individuals receive timely diagnoses, services and interventions. Good assessments combined with adapted programs and initiatives provide essential support to individuals and their families, addressing their unique needs and circumstances. Our frontline intervention capacity and direct proximity with communities support and enrich knowledge exchanges from lived experiences to policy makers, researchers and practitioners. Real‐life stories and testimonies highlight the positive impact of these initiatives, showcasing how community health efforts significantly enhance the quality of life for individuals with neurocognitive disorders and their care partners.

In conclusion, the leadership of our community health organization in initiating a collaborative group and the shared strategic alignment has been instrumental in driving meaningful changes. By fostering a multidisciplinary approach, intersectorial collaboration and leveraging the strengths of policy, clinical practice, and community sectors, we have created a robust framework for improving care pathways and quality of life.

